# Jean St. Germain, M.S. 

**DOI:** 10.1002/acm2.12273

**Published:** 2018-02-03

**Authors:** Lawrence Rothenberg, Lawrence Dauer

**Affiliations:** ^1^ New York NY

It is with great sadness that we share that Jean St. Germain, our colleague, friend, and integral member of the Medical Physics Department at Memorial Sloan Kettering Cancer Center (MSKCC) for over 50 yr, passed away on December 7, 2017. She died peacefully and was with her brother, Amos, at the time.

Following completion of graduate study at Rutgers University and a fellowship at Brookhaven National Laboratory, Jean was appointed, in November 1967, as a Fellow in the Department of Medical Physics at Memorial Sloan Kettering under John Laughlin and Garrett Holt. At the end of her fellowship, she was appointed to the faculty and rose to the rank of Associate Attending Physicist, and subsequently, to Attending Physicist. She served as the Corporate Radiation Safety Officer, guiding and presiding over the incredible growth of the institution. She served as an interim chair of the Department of Medical Physics from 2007 to 2010 and subsequently as a Vice‐Chair for Clinical and Educational Affairs and Clinical Member. Jean was a licensed medical physicist in New York State and was certified in Comprehensive Health Physics in 1974 by the ABHP and in 1991 in Medical Health Physics by the ABMP. Jean was also appointed a Lecturer, Instructor, and ultimately Assistant Professor of Physics in Clinical Radiology, Weill College of Medicine, Cornell University and served as the Radiation Safety Officer at the NY Presbyterian Weill Cornell Medical Center for more than 35 yr.

Jean St. Germain 1945–2017

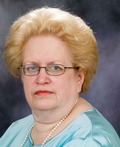

Jean's contributions to the field of medical physics and health physics were vast and significant. She has served several professional societies in key leadership roles. She served AAPM as National Secretary, Chair of the Rules Committee, Parliamentarian, founding Chair of the Development Committee, Member of the Governing Board of the AIP, Treasurer of the American Academy of Health Physics, Chair of the Examining Panel in Medical Health Physics and Vice‐Chair of the American Board of Medical Physics. She has served four terms on the AAPM Board of Directors. In the Greater New York area, she served as the President of the Radiological and Medical Physics Society (RAMPS, the NYC Chapter of the AAPM) and served three terms as the President of the Greater NY Chapter of the HPS.

Jean was a member of the Scientific Committee (SC) for the National Council on Radiation Protection and Measurements (NCRP) that produced NCRP Report No. 105 on radiation protection of medical and allied health personnel. Jean later served as the Chairman of the SC that produced NCRP Report No. 155 on the management of radionuclide therapy patients. In addition, Jean served as a member of several New York State advisory committees on medical and radiological health. She also served as a special examiner for the New York State Civil Service Commission.

Jean received many honors and awards during her career. She was a Fellow of the Health Physics Society and of AAPM. She was presented the Failla Award by the Greater NY Chapter HPS and RAMPS. She received the AAPM Distinguished Service Award in 2001 as well as the Varian Award for best professional paper in the Journal of Applied Clinical Medical Physics in 2004. And in 2015, Jean was presented with the Marvin M. D. Williams Professional Achievement Award by the AAPM. The award recognizes AAPM members for an eminent career in medical physics with an emphasis on clinical medical physics.

Jean was an excellent lecturer and teacher. She taught Health Physics and Radiation Safety to generations of medical physicists, radiologists, radiation oncologists, nuclear medicine physicians, radiotherapists, radiologic technologists, lab scientists, and others at MSKCC, Weill Cornell Medical Center and throughout the medical physics and radiological community.

Beyond physics, Jean's great passion was music. She took vocal lessons at Julliard and was an operatic soprano soloist who gave many recitals and concerts throughout her life. She was also a regular attendee of performances at the Metropolitan Opera. Her commitment to service extended to her church, St. Joseph's in Yorkville, where she was a Trustee. She was also an active member of the National Society of Arts and Letters, serving on the Winston Scholarship Committee, and the Shirley Rabb Winston Scholarships in Voice.

Jean considered Marie Curie a heroine as she was the first woman to win a Nobel prize and then became the first person to attain a second Nobel prize. Jean had the pleasure of spending time with Marie Curie's daughter, Eve Curie Labouisse, who had written an extensive biography of her mother.

She is survived by her brother, Amos St. Germain, his wife, Susan, and their three children. Jean was particularly delighted with her two grand‐nieces, Natalie and Maren, and shared photos of them with her colleagues at every opportunity.

In lieu of flowers, donations may be made to the Memorial Sloan Kettering Cancer Center – John Laughlin Research & Education Fund, in memory of Jean St. Germain. The website is www.mskcc.org, click on the ‘Giving’ tab. For additional information, please contact Wei Lui, MSKCC, Department of Medical Physics, 1275 York Ave., New York, NY 10065 (lui1@mskcc.org).

Jean was an accomplished person of wide‐ranging interests and service, with a distinct sense of professionalism and honor, who was engaged with the world and loved by friends and family members. She will be greatly missed.

